# A digital mindfulness intervention improves sleep efficiency and heart rate variability in healthy adults

**DOI:** 10.1038/s41598-026-44902-w

**Published:** 2026-03-21

**Authors:** Ulrich Kirk, Cirkeline Nellemann Hovgaard, Marino Theodor Larsen Persiani, Marco Romagnoli, Walter Staiano

**Affiliations:** 1 Department of Psychology, University of Southern Denmark, 5230 Odense, Denmark; 2 Fralin Biomedical Research Institute at VTC, Virginia Tech, Roanoke, VA USA; 3 Department of Physical Education and Sport, University of Valencia, 46010 Valencia, Valencia Spain

**Keywords:** Wearable data, Mindfulness, Stress, Oura, Headspace, Sleep, Translational research, Human behaviour

## Abstract

To test whether a 10-day mindfulness program delivered through the ŌURA app improves sleep and stress in healthy adults, eighty-one adults were randomized to mindfulness (n = 49) or waitlist control (n = 32). Participants wore an Ōura ring during baseline, intervention/wait-list, and 4-week follow-up, recording sleep efficiency, total, deep, and light sleep, plus sleep-onset time. Questionnaires—Pittsburgh sleep quality index (PSQI), perceived stress scale (PSS), Copenhagen burnout inventory (CBI), and mindful attention awareness scale (MAAS)—were completed pre- and post-intervention and at follow-up. Mixed-model ANOVAs revealed significant group × time interactions for sleep efficiency, total sleep, deep and light sleep, and sleep-onset time (all *p*s < 0.031). Results showed that the mindfulness group improved after 10 days (all *p*s < 0.021); gains persisted at follow-up except for deep sleep. The mindfulness group exhibited increased personal burnout (*p* = 0.021) immediately post-intervention, though this returned toward baseline at follow-up. In addition, the mindfulness showed higher MAAS scores (*p* = 0.017). During mindfulness sessions heart rate fell (*p* = 0.011) and heart-rate variability rose (*p* = 0.029). A brief, app-based mindfulness program produced sustained improvements in sleep efficiency and enhanced HRV, demonstrating that digital mindfulness can favorably influence biobehavioral sleep-stress metrics.

*Trial Registration*: ClinicalTrials.gov: NCT07469644

## Introduction

Sleep is essential for optimal functioning and overall well-being, yet millions worldwide continue to suffer from poor sleep quality and sleep disorders. Disruptions in sleep are frequently observed in psychiatric conditions^1–3^, and it is indicated that up to 70 million Americans^4^ and over 31% of the general population in the Netherlands^5^ experience such disturbances. These challenges underscore the need for effective, scalable interventions to improve sleep and enhance health outcomes, with potential clinical applications that extend beyond research contexts to everyday therapeutic practices.

Mindfulness, characterized by focused and non-judgmental awareness of present-moment experiences, has consistently been associated with improvements in sleep-related outcomes by targeting key cognitive and emotional mechanisms. Mindfulness practices have been shown to effectively reduce maladaptive processes such as rumination^6^ and emotional reactivity^7^, both of which are closely linked to sleep disturbances. A substantial and growing body of research supports the efficacy of mindfulness-based interventions in improving sleep quality across both clinical and non-clinical populations^8–11^. These approaches not only enhance subjective sleep experience but also contribute to measurable physiological benefits. For example, our previous work^12^ demonstrated that a brief 10-day online mindfulness program significantly increased heart rate variability (HRV) both during mindfulness sessions and during sleep, indicating improvements in autonomic regulation that may support better sleep architecture. This physiological link has been further corroborated by Balsam et al.^13^, who reported significant shifts in HRV metrics following mindfulness training in a cohort of pregnant women—a population at heightened risk for sleep difficulties.

Recent advances in wearable technology offer new methodological tools for examining the physiological effects of behavioral interventions on sleep. Devices capable of continuously monitoring heart rate variability, sleep architecture, and other autonomic markers enable objective, longitudinal assessment of intervention outcomes. Beyond passive data collection, these tools allow for the exploration of within-person dynamics, such as how day-to-day variations in mindfulness practice may relate to physiological recovery and sleep quality. Integrating such biometrics into intervention research supports a more precise understanding of the mechanisms linking mindfulness to sleep regulation.

Several scalable digital solutions are currently available to deliver mindfulness strategies including popular Apps such as Ōura (Oulu, Finland). On the other hand, the rise in wearable technology like the ŌURA Ring enabled passive longitudinal real-time assessments and user feedback on sleep, cardiorespiratory data and other health metrics in free-living conditions, empowering self-monitoring and behavioral changes (e.g., keep regular bedtimes). Longitudinal monitoring capabilities are particularly valuable for examining not only immediate intervention effects but also their sustainability and potential decay curves over time, helping to determine which sleep parameters require continued practice to maintain improvements. Integrating digital mindfulness interventions with wearable technology could facilitate therapeutic access as well as boosting effectiveness by introducing real-time feedback on biobehavioral data—such as heart rate variability (HRV) and sleep—that has been shown to be sensitive to changes induced by mindfulness practices.

In the present randomized study, we combined in-app mindfulness intervention with biobehavioral tracking and feedback using the Ōura ring. The primary aim was to explore the potential of combining digital therapeutics with health monitoring to produce sustainable improvements in sleep quality and autonomic function. Specifically, we seek to clarify whether mindfulness practice—delivered digitally—can improve key parameters of sleep initiation and maintenance, particularly sleep onset time, which represents one of the most common complaints among individuals with sleep disturbances^14^. This approach not only builds upon our previous findings but also aims to establish a framework for future digital health interventions targeting sleep-related issues. Our multi-method approach incorporating both objective and subjective sleep measures allowed us to examine potential discrepancies between physiological improvements and perceived changes in sleep quality—a phenomenon previously observed in mindfulness research that may reflect the complex relationship between measurable sleep parameters and subjective sleep experience.

## Methods

### Participants

**Fig. 1:**
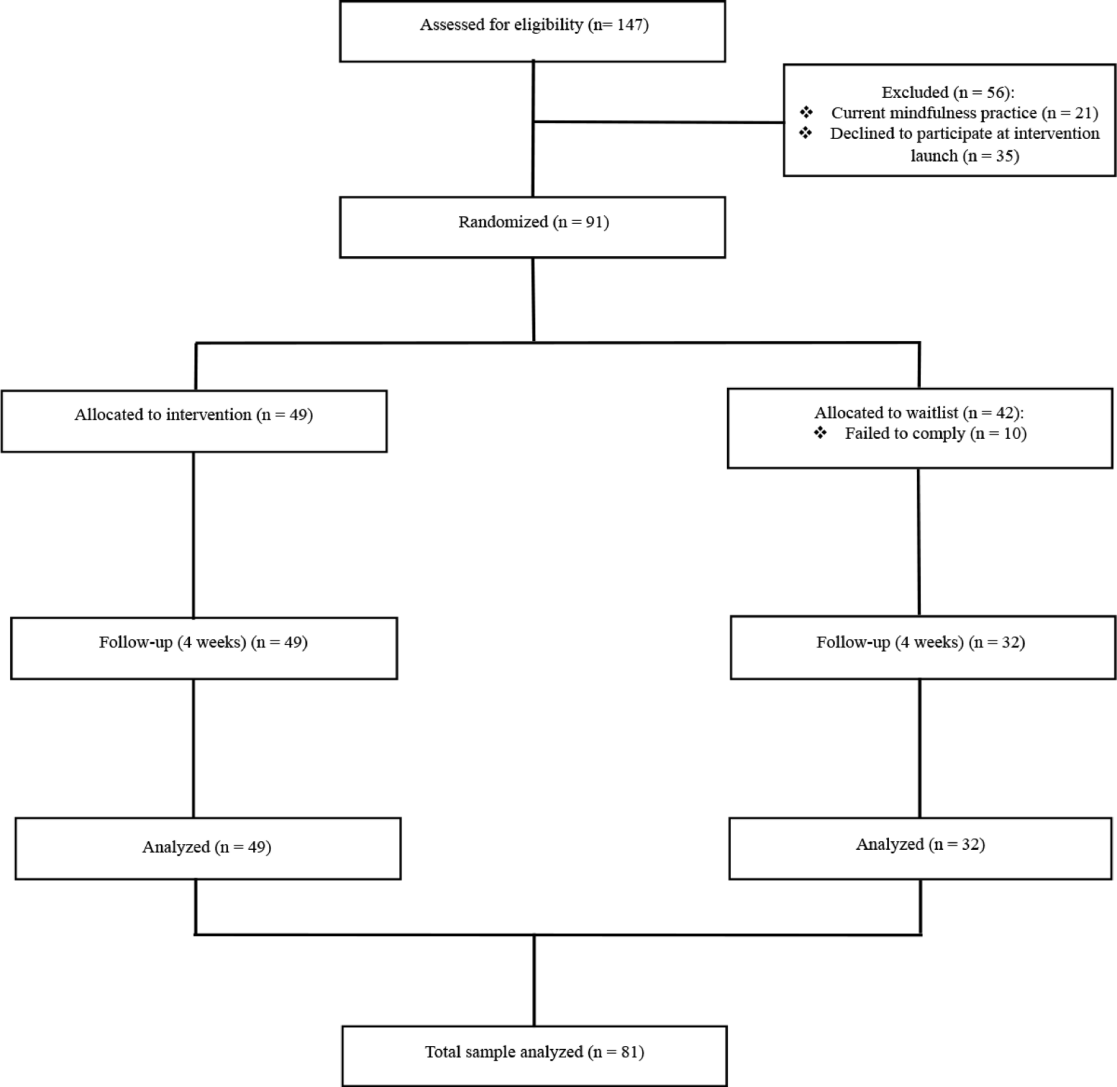
CONSORT diagram.

A total of 91 healthy participants were recruited through flyers and advertisements at a local university (University of Southern Denmark) and the Region of Southern Denmark and randomly assigned to two study groups: mindfulness intervention and waitlist control. Ten participants in the control group were excluded due to a lack of adherence with the protocol leading to a final sample of 81 participants (Fig. 1). Demographics and sample characteristics are provided in Table 1.

**Table 1:** Demographics and compliance by study group.

	Mindfulness (N = 49)	Waitlist Control (N = 32)
Gender, n (%)		
Male	19 (38.8)	16 (50.0)
Female	30 (61.2)	16 (50.0)
Age, mean (SD)	25.50 (0.67)	25.13 (1.4)
BMI, mean (%)		
18.5–24.9	35 (69.3)	20 (62.5)
25–29.9	14 (20.5)	12 (31.4)
Occupation, n (%)		
Student	40 (81.6)	22 (68.8)
Fulltime employed	3 (6.1)	1 (3.1)
Multiple jobs	–	1 (3.1)
Parttime employed	2 (4.1)	4 (12.5)
Self-employed	–	2 (6.3)
Unemployed	2 (4.1)	1 (3.1)
Other	2 (4.1)	1 (3.1)
Ethnicity, n (%)		
White	46 (93.9)	26 (81.3)
Asian	–	–
Black	2 (4.1)	1 (1.3)
Hispanic	1(2.0)	–
Middle Eastern	–	3 (9.4)
Other	–	2 (6.3)
MEQ, n (%)		
Evening types	18 (36.7)	10 (31.3)
Intermediate types	27 (55.1)	21 (65.6)
Morning types	4 (8.2)	1 (3.1)
Compliance rate, (no of sessions/10 sessions in total) (SD)	7.6 (3.6)	–
Sleep records (nights), Mean ± SD		
Pre-intervention	6.8 (0.4)	6.7 (0.5)
Post-intervention	6.7 (0.5)	6.6 (0.6)
Follow-up	6.6 (0.6)	6.5 (0.7)

Inclusion criteria for the study were healthy adults in the age 20–60 years, with a body mass index (BMI) in the normal range 18.5–29.9 kg/m^2^. Exclusion criteria were current regular practice of mindfulness (> 20 min per week), self-reported diagnosis of cardiovascular disease, self-reported history of neurodegenerative diseases, self-reported current diagnosis of anxiety or depression or use of medications.

All procedures involving human participants were approved by the Regional Committee on Health Research Ethics for Southern Denmark (Videnskabsetisk Komité for Region Syddanmark), Approval ID: S-20232000-78. The study was conducted in full accordance with the ethical principles of the Declaration of Helsinki (2013 revision). Written informed consent was obtained from all participants prior to enrolment.

Participants received compensation for their participation in the study, specifically they were given the Ōura ring if they completed the study. Recruitment took place from December 2023 to April 2024.

Power analysis indicated that a sample size of 81 (total number of participants minus 10–12% calculated dropout) would provide 80% power to detect significant (*p* < 0.05) between-within interaction effects (f = 0.29, η^2^p = 0.08).

### Randomization and attrition

Participants were initially randomized using a computer-generated random sequence to achieve balanced group allocation between the mindfulness intervention and waitlist control conditions. However, the final sample size reflected differential attrition between groups occurring after randomization. Ten participants initially assigned to the waitlist control group were excluded post-randomization due to non-adherence with study protocols (defined as failure to wear the Ōura ring for more than 3 consecutive days or wearing it for less than 70% of the required study period). No participants in the mindfulness intervention group were excluded for non-adherence, resulting in the final unbalanced sample (n = 49 mindfulness, n = 32 control). This differential attrition pattern may reflect differences in engagement and motivation between active intervention and waitlist conditions, representing a limitation in our study design.

### Study design and timeline

This mindfulness intervention study was embedded within a larger 4-month longitudinal investigation examining physiological stress and sleep quality. The overall study design included: (1) a 1-month baseline period during which participants wore the Ōura ring continuously to establish individual physiological baselines, (2) a 3-month free-living period during which various sub-studies were conducted, including the present 10-day mindfulness intervention. The mindfulness intervention reported in this manuscript was conducted approximately 2 months after initial enrollment, ensuring that all participants had extensive baseline physiological data (> 60 days) before the intervention period. This extended baseline period provided robust individual-level baselines for sleep and autonomic measures, well exceeding the ~ 28-day period typically required for accurate wearable-based HRV assessment.

The larger study enrolled 140 participants stratified by stress levels (50% high stress PSS ≥ 27, 50% low stress PSS ≤ 13). The current analysis focuses specifically on the low-stress participants (n = 81 after exclusions) who participated in the mindfulness intervention sub-study during the broader investigation. This design choice allowed us to examine mindfulness effects in individuals without pre-existing high stress levels, enabling detection of more subtle improvements and examination of mindfulness as a preventive rather than therapeutic intervention. While this approach provides valuable insights into mindfulness effects in relatively healthy populations, we acknowledge it represents a limitation for generalizability to clinical populations with significant stress or sleep disorders.

### Experimental procedures

Potential participants were invited to come to the lab for an initial visit in which they gave written consent to participate in the study and completed the MEQ (Morningness-Eveningness Questionnaire (MEQ)^15^. Participants were also instructed about the study’s logistics and procedures for the remote testing. They were told how to use the Ōura ring and navigate and use the Ōura in-app mindfulness intervention. Participants were asked to wear the Ōura ring daily for the entire duration of the study. Participants did not have visibility into the Daystress stress metric for the duration of the study. The experimenters monitored the Ōura server throughout the study period to ensure compliance with wearing the ring. Participants were instructed to fulfill electronic surveys before and after the 10-day mindfulness or waitlist control intervention, and at the 4-week follow up using an online-based platform (www.survey-xact.dk). The stats and trends displayed in the Ōura app were visible to participants in both groups during the study. Participants were allocated to either a mindfulness group or a waitlist control group in a random manner. Sequence generation and randomization was performed by the research team, who were not formally blinded to group allocation. Participants were informed that they would be randomly assigned to one of the two groups, which eliminated any self-selection bias across the intervention groups.

### Intervention

#### Mindfulness

All participants were given access to the Ōura app (https://support.ouraring.com). The participants in the intervention group were instructed to complete daily mindfulness practices available in the ŌURA app in the Explore tab. Specifically, the participants were asked to complete the session entitled ‘destress’, which was provided by Headspace (www.headspace.com). The exercise had a duration of 10 min. Participants were asked to complete the mindfulness session once per day at any time during the day for a period of 10 days. It is noteworthy that the ‘destress’ mindfulness exercise targeted stress reduction and not sleep. Compliance was provided by a function in the app that tracked the timestamps to keep count of content length on a subject-by-subject basis (Table 1).

#### Waitlist

The waitlist control group required that participants do not follow an intervention. However, the waitlist control group had access to mindfulness content after completion of the study.

### Follow-up period procedures

Following completion of the 10-day intervention or waitlist period, all participants entered a 4-week follow-up phase to assess sustainability of any intervention effects. During this period, participants in both groups continued wearing the Ōura ring daily for continuous collection of sleep and physiological data. No active interventions were provided during follow-up. Participants in the mindfulness group were specifically instructed to refrain from using any mindfulness content available in the Ōura app (including Headspace sessions) during the entire 4-week follow-up period to ensure that any observed effects reflected sustained benefits from the initial 10-day intervention rather than continued practice. Compliance with this instruction was monitored through app usage logs. Self-report questionnaires (PSS-10, PSQI, CBI) were administered at the end of the 4-week follow-up period to assess longer-term changes in perceived stress, sleep quality, and burnout. The follow-up period allowed examination of whether brief mindfulness training produces lasting physiological and behavioral changes or whether benefits require ongoing practice to maintain.

### Compliance and data quality

Compliance monitoring was implemented to ensure data quality and protocol adherence across all study phases. Ōura ring wear time was monitored continuously through the device’s server connectivity, with both groups achieving excellent compliance (> 90% wear time throughout the 4-month study period). For the mindfulness intervention group, session completion was tracked through the app’s built-in logging system. Self-report survey completion rates exceeded 85% for all timepoints across both groups (pre-intervention: 89%, post-intervention: 87%, follow-up: 86%). During the 4-week follow-up period, app usage was continuously monitored to ensure participants in the mindfulness group refrained from accessing mindfulness content. Compliance with this instruction was 100%, with no recorded usage or other mindfulness features during the follow-up phase.

### Self-report surveys

#### Perceived stress scale (PSS-10)

The PSS-10 consists of ten items designed to measure self-perceived stress^16^. Participants answer questions about their feelings and thoughts using a 5-point Likert scale (0 = never to 4 = very often). Scores range from 0 to 40 with higher scores indicating more stress. The scale has high test–retest reliability (Cronbach’s α = 0.85).

#### The Pittsburgh sleep quality index (PSQI)

The PSQI is a self-rated questionnaire which assesses sleep quality^17^. Nineteen individual items generate seven component scores: subjective sleep quality, sleep latency, sleep duration, habitual sleep efficiency, sleep disturbances, use of sleeping medication, and daytime dysfunction. The sum of scores for these seven components yields one global score^17^. Higher scores indicate worse sleep quality whereas poor sleep will be a PSQI total score of > 5^17^.

#### Copenhagen burnout inventory (CBI)

CBI is a validated survey^18^ and consists of thirteen questions from two dimensions: (a) Personal burnout and (b) Work-related burnout. CBI1 refers to the personal burnout subscale, which measures the degree of physical and psychological fatigue and exhaustion experienced by the individual regardless of occupational context. CBI2 refers to the work-related burnout subscale, which measures the degree of physical and psychological fatigue and exhaustion that is perceived by the individual as related to their work. Response categories are Always, Often, Sometimes, Seldom, Never/almost never and to a very high degree, To a high degree, Somewhat, To a low degree, To a very low degree. Scoring used is as follows: always/to a very high degree = 100%, Often/ to a high degree = 75%, Sometimes/Somewhat = 50%, Seldom/ to a low degree = 25%, and never/almost never/to a very low degree = 0%. An average score is generated from all these 13 questions and burnout score is categorized as follows: ≤ 25 (no burnout), 26–50 (moderate burnout), and > 50 (high burnout).

#### Mindful attention awareness scale (MAAS)

The MAAS is a 15-item scale designed to assess trait or dispositional mindfulness^19^. Specifically, the MAAS measures attention to and awareness of the present moment. It has good psychometric qualities (Cronbach’s α = 0.89). The 15 items are rated on a 6-point Likert scale. The MAAS has a range of 15–90 points and is scored by calculating the sum of the items and dividing by the total numbers of items. Higher scores indicate higher levels of trait mindfulness.

In this study we only administered the MAAS in the intervention group, but not in the waitlist control group in that we wanted to ascertain if the intervention impacted mindfulness.

#### Morningness-eveningness questionnaire (MEQ)

The Morningness-Eveningness Questionnaire (MEQ)^15^ is the most commonly used circadian typology questionnaire and consists of 19 questions with Likert responses about an individual’s preferred bedtimes, raise times, and times for activity. Responses to questions are scored and summed to produce an overall morningness score ranging from 16 to 86, with higher scores indicating greater morningness^15^.

#### Ōura ring biobehavioral measures

Biobehavioural data were collected by the Ōura ring (Gen 3), which wirelessly syncs data to the ŌURA app and server. The Ōura ring uses photoplethysmography. ŌURA ring measurements of HRV [more specifically: Square root of the mean of the squares of successive NN interval differences (RMSSD) measured in ms] and other physiological metrics have previously been validated for accuracy relative to electrocardiography^20^. Sleep data were collected by measuring resting heart rate (HR), body temperature, time (min and in percentage) spent in specific sleep stages (including light, deep, and rapid eye movement), and movement via actigraphy, a validated method of measuring sleep via an accelerometer^21^. For the in-session mindfulness training we extracted the 10 min mindfulness session for each of the 10-day intervention period and averaged HR and HRV data across the number of sessions per participant.

For the analysis of acute physiological effects during mindfulness practice, we extracted heart rate (HR) and heart rate variability (HRV) data during the 10-min mindfulness sessions for each day of the intervention period. To establish an appropriate comparison baseline for these in-session measurements, we collected HR and HRV data during equivalent 10-min periods immediately preceding each mindfulness session (same time of day, same participant conditions). This pre-session baseline allowed us to assess the acute physiological changes specifically attributable to mindfulness practice by comparing within-participant HR and HRV values during active mindfulness versus the immediately preceding rest period. Data were averaged across all completed sessions per participant. This enabled detection of acute autonomic changes occurring during mindfulness practice while controlling for individual differences in baseline physiology and time-of-day effects on cardiovascular parameters.

To ensure standardized measurement periods across participants, we established precise definitions for all study timepoints. Pre-intervention measurements were calculated by averaging sleep and physiological data across the 7 days immediately preceding the start of the 10-day intervention period. Post-intervention measurements were calculated by averaging data across the 7 days immediately following completion of the intervention period. Follow-up measurements were calculated by averaging data across the final 7 days of the 4-week follow-up period (days 22–28 of follow-up). This 7-day averaging approach was implemented to minimize the influence of night-to-night variability in sleep patterns and provide stable, representative measures for each study phase. For participants who had missing data on specific nights due to device malfunction or non-wear, timepoint averages were calculated using available valid nights within each 7-day window, provided at least 5 valid nights of data were available per timepoint.

### Statistical analysis

Assumptions of statistical tests for normal distribution and sphericity of data were checked. A series of mixed 2 group (mindfulness intervention, waitlist control) × 3 timepoints (pre-intervention, post-intervention, follow-up) ANOVAs were performed on sleep (Sleep period duration [min], Time Asleep [min], Time spent in Deep Sleep [% and min], REM Sleep [% and min], Light Sleep [% and min], Sleep Onset Time [min]) and sleep HR and HRV), and surveys (PSS-10, PSQI, CBI 1 and 2). A series of paired T-Tests were performed in the mindfulness group only for MAAS at pre-intervention and post-intervention; and average HR and HRV variables during each mindfulness session and the average HR and HRV before each mindfulness session. All significant interactions were followed up with relevant corrected pairwise comparisons using the Bonferroni method for simple main effects within each group. Where no significant interactions were found, main effects of time and group were reported. Significance was set at 0.05 (2-tailed) for all analyses and ANOVA effect sizes were calculated as partial eta squared (η^2^p) with 0.02, 0.13, and 0.26 indicating small, medium, and large effects, respectively. For T-tests effect size was calculated using Cohen`s d. Data analysis was conducted using SPSS (version 27; IBM Corp).

## Results

All results (mean, SD, *p* values and effect sizes) are reported in Table 2.

**Table 2:** Outcome variables employed in the study at pre, post and follow-up.

Measures	PRE		POST		Follow-up		Time		Group		Group × time	
	MI	CON	MI	CON	MI	CON	*p*	ηη_p_^2^ − d	*p*	η_p_^2^	*p*	η_p_^2^
Sleep												
Sleep onset time (min.)	26 ± 55	16 ± 61	− 2 ± 51	25 ± 59	− 14 ± 61	35 ± 59	0.429	0.012	0.168	0.029	0.008*	0.079
Sleep efficiency (%)	87.7 ± 4.2	87.7 ± 4.1	88.72 ± 3.4	85.5 ± 4.6	89.06 ± 3.5	87.84 ± 4.5	0.023*	0.048	0.147	0.026	0.031*	0.044
Total sleep (min.)	448 ± 45	448 ± 49	463 ± 44	431 ± 59	457 ± 54	445 ± 55	0.759	0.003	0.139	0.027	0.010*	0.055
Time in bed (min.)	551 ± 47	515 ± 61	522 ± 53	499 ± 81	516 ± 64	507 ± 63	0.950	0.001	0.433	0.008	0.129	0.025
Sleep latency (min.)	18.4 ± 9.6	19.2 ± 9.2	17.3 ± 9.5	20.2 ± 9.2	16.4 ± 9.6	17.8 ± 8.6	0.201	0.020	0.332	0.012	0.627	0.006
Deep sleep (min.)	91.2 ± 16.7	89.1 ± 21.2	95.6 ± 22.6	85.41 ± 23.1	90.66 ± 18.1	87.03 ± 18.4	0.512	0.008	0.207	0.020	0.022*	0.048
REM sleep (min.)	104.3 ± 20	100.2 ± 20	105.4 ± 19	97 ± 21	103.9 ± 21	99.2 ± 21	0.846	0.002	0.175	0.023	0.495	0.009
Light sleep (min.)	252.7 ± 29	258.1 ± 45	263.8 ± 31	247.9 ± 52	263.3 ± 37	258.9 ± 49	0.255	0.017	0.531	0.005	0.023*	0.046
Deep sleep (%)	20.35 ± 3.6	20.05 ± 5.1	20.59.6 ± 4.9	20.16 ± 5.5	20.01 ± 4.8	19.85 ± 4.4	0.626	0.003	0.732	0.002	0.844	0.001
REM sleep (%)	23.07 ± 3.4	22.29 ± 3.4	22.58 ± 3.1	22.38 ± 3.4	22.55 ± 3.3	22.45 ± 3.6	0.491	0.006	0.506	0.006	0.317	0.014
Light sleep (%)	56.54 ± 4.3	57.44 ± 7.1	57.28 ± 4.7	57.27 ± 7	57.68 ± 5.1	57.91 ± 6.9	0.514	0.006	0.725	0.002	0.300	0.014
Physiological												
HR	59.7 ± 6.9	59.2 ± 9.3	59.7 ± 7.5	59.5 ± 8.1	59.9 ± 7.8	59.9 ± 8.3	0.607	0.006	0.889	0.000	0.853	0.002
HRV	70.5 ± 30.4	66.7 ± 32.5	70.9 ± 31.4	65.4 ± 35.5	69.8 ± 31	65.4 ± 30.8	0.827	0.002	0.511	0.005	0.871	0.002
Respiratory Fr	15.4 ± 1.4	15.2 ± 1.4	15.3 ± 1.5	15.2 ± 1.2	15.4 ± 1.5	15.2 ± 1.3	0.504	0.008	0.611	0.003	0.870	0.002
Self-report												
PSS	13.5 ± 6.8	11.9 ± 8.2	14.7 ± 6.1	11.9 ± 7.2	12.1 ± 6.4	13.3 ± 8.7	0.251	0.022	0.187	0.023	0.613	0.005
PSQI	9.2 ± 2.7	9.0 ± 3.2	9.2 ± 2.5	9.2 ± 3.5	8.8 ± 2.6	9.7 ± 2.8	0.687	0.002	0.926	0.000	0.692	0.001
CBI 1	38.2 ± 21	38.3 ± 22	42.4 ± 17	32.5 ± 21	38.8 ± 19	35.9 ± 19	0.552	0.003	0.313	0.013	< 0.01*	0.128
CBI 2	37.7 ± 15	37.9 ± 17	39.3 ± 15	34.8 ± 14	36.6 ± 15	35.3 ± 13	0.571	0.006	0.532	0.005	0.099	0.006
MAAS	3.8 ± .74		4.1 ± .66				0.017*	0.356				

### Timing of mindfulness practice

Analysis of app usage timestamps revealed that participants practiced mindfulness at various times throughout the day. The majority (58%) practiced between 7 and 10 PM (evening), 23% between 6 and 9 AM (morning), 12% between 12 and 3 PM (midday), and 7% at other times. A sensitivity analysis examining sleep outcomes by timing of practice found no significant differences in sleep improvements across timing groups (sleep efficiency: F(3,45) = 0.89, *p* = 0.45; total sleep time: F(3,45) = 1.12, *p* = 0.35; sleep onset time: F(3,45) = 0.76, *p* = 0.52), indicating that mindfulness practice benefits sleep regardless of when during the day it is performed.

### Sleep

**Fig. 2:**
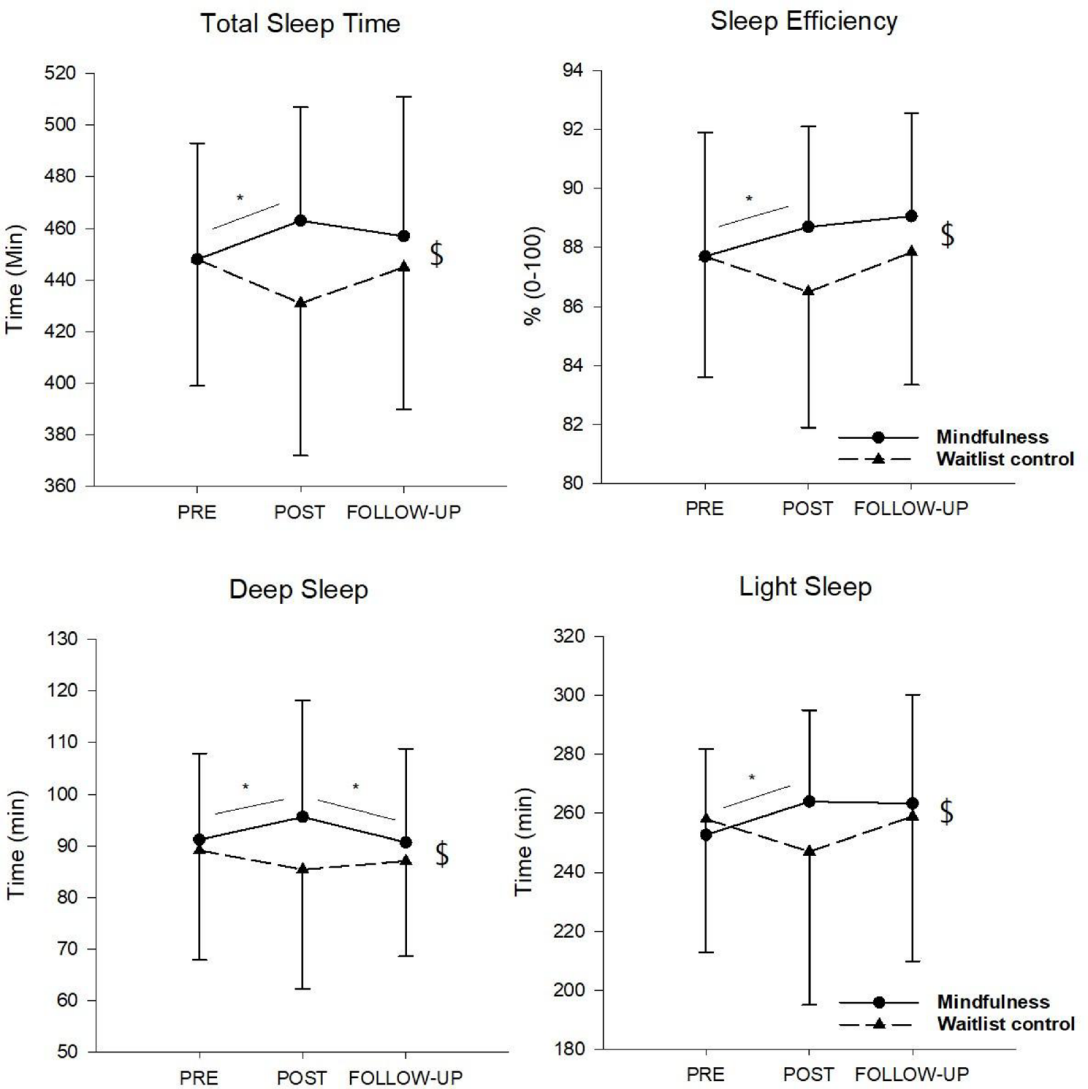
Sleep variables at pre, post and follow-up for the mindfulness and waitlist control groups. Data are presented as mean (SD). $ = significant condition × time interaction. * = significant simple main effect.

Sleep efficiency, total sleep, deep sleep (in minutes), light sleep (minutes) and sleep onset time (Fig. 2) showed significant group by time interactions. Follow up tests revealed that the mindfulness training improved these sleep variables from pre to post while no significant differences were reported in the control group. Moreover, at 4-week follow-up it was shown that the mindfulness group maintained sleep improvements, with exception of deep sleep (in minutes) which returned to baseline levels. No significant interactions or main effects were reported for time in bed, sleep latency, REM sleep and deep and light sleep.

### Physiological

#### Resting physiological measures across study phases

Resting heart rate (HR), heart rate variability (HRV), and respiratory frequency measured during sleep did not show significant changes in either group from pre to post to follow-up, indicating that the intervention did not produce sustained changes in resting nocturnal autonomic parameters over the study period.

#### Acute physiological changes during mindfulness sessions

**Fig. 3:**
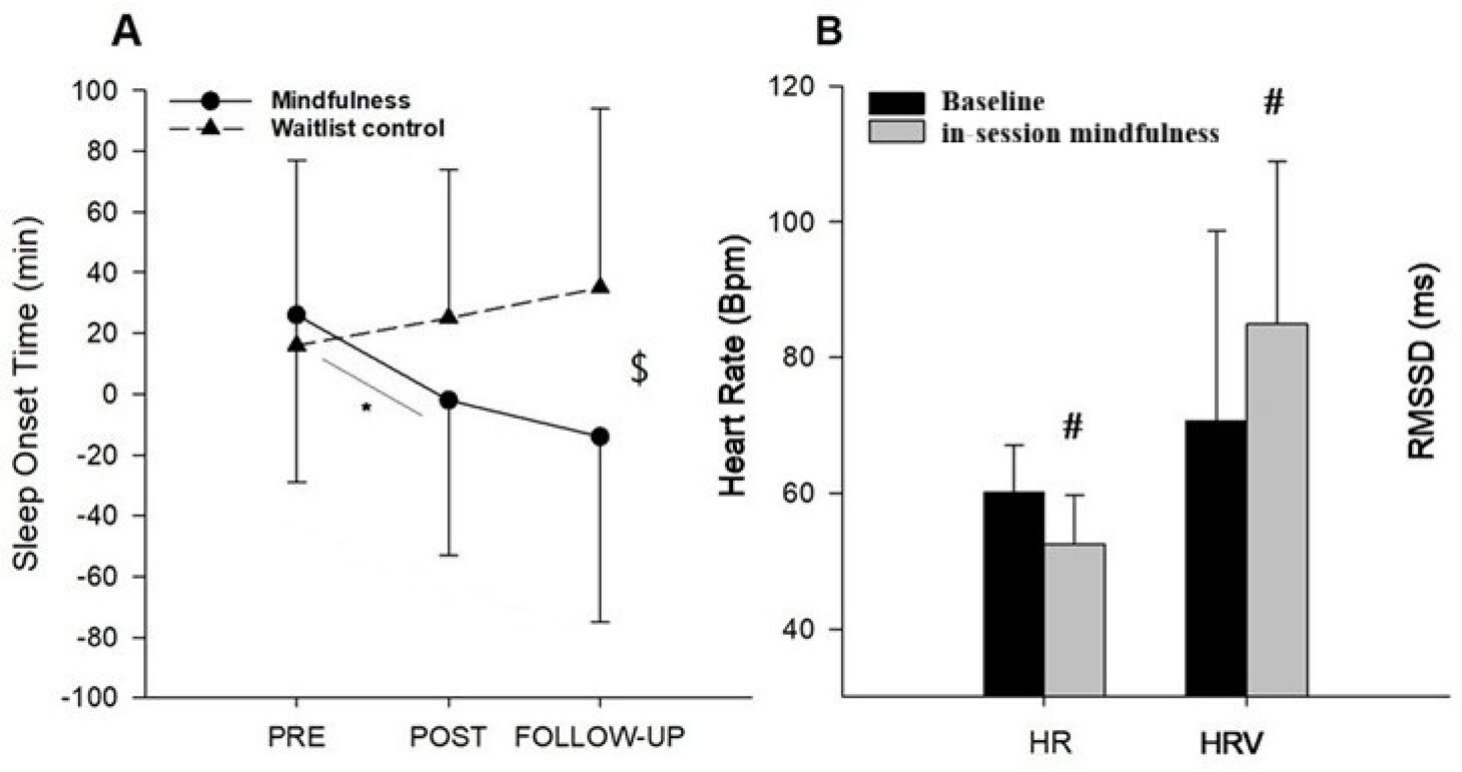
Sleep onset time and in-session cardiac activity. (**A**) Sleep onset time measured at pre, post and follow-up in mindfulness and waitlist control group. (**B**) Physiological activity represented as heart rate (HR) measured in beats per minute (bpm) and heart rate variability (HRV) measured as root mean square of successive differences between normal heartbeats (RMSSD) during mindfulness session and during a similar time-period when participants were not practicing mindfulness. Data are presented as mean (SD). $ = significant condition × time interaction. # = significant difference between conditions (*p* < 0.05). * = significant simple main effect (*p* < 0.05).

In contrast, when examining within-session effects during the 10-day intervention period, average HR decreased significantly (t(48) = 2.63, *p* = 0.011, Cohen’s d = 0.45) and HRV increased significantly (t(48) = 2.24, *p* = 0.029, Cohen’s d = 0.38) in the mindfulness group when comparing values during the 10-min mindfulness sessions to equivalent 10-min baseline periods immediately preceding each session (Fig. 3). These acute changes indicate parasympathetic activation and reduced physiological arousal during mindfulness practice.

### Self-report surveys

No significant group by time interactions or main effects were reported for PSS, PSQI and CBI2. However, the mindfulness intervention resulted in a significant increase in CBI1 (personal burnout subscale) from pre to post, while no significant changes were observed in the waitlist control group. At the 4-week follow-up, CBI1 scores in the mindfulness group showed a non-significant trend toward returning to baseline levels. In the mindfulness group, there was a significant increase in MAAS scores from pre to post (*p* < 0.017), indicating increased dispositional mindfulness following the intervention.

### Sensitivity analyses

Student subsample analysis: A sensitivity analysis restricted to student participants only (n = 40 mindfulness vs. n = 22 control) showed results consistent with the full sample: significant group × time interactions for sleep efficiency (F(2,120) = 3.28, *p* = 0.041, ηp^2^ = 0.08), total sleep time (F(2,120) = 3.35, *p* = 0.038, ηp^2^ = 0.08), deep sleep (F(2,120) = 3.18, *p* = 0.045, ηp^2^ = 0.08), and sleep onset time (F(2,120) = 3.52, *p* = 0.034, ηp^2^ = 0.09). Effect sizes were comparable to the full sample analysis, suggesting findings are not driven solely by student versus non-student differences.

Weekday/weekend analysis: Post-hoc analysis including weekday/weekend as a covariate showed a significant main effect of day-of-week (F(1,79) = 4.87, *p* = 0.030), with participants sleeping approximately 23 min longer on weekends. However, group × time interactions remained significant when controlling for this factor (sleep efficiency: F(2,158) = 3.67, *p* = 0.028, ηp^2^ = 0.09; total sleep time: F(2,158) = 3.89, *p* = 0.023, ηp^2^ = 0.10; sleep onset time: F(2,158) = 4.21, *p* = 0.017, ηp^2^ = 0.11).

## Discussion

The study pursued a twofold aim: to replicate earlier findings that an app-based mindfulness intervention can acutely increase heart rate variability (HRV) during sessions—indicating reduced physiological arousal^12^—and to assess whether these immediate effects translate into sustained improvements in sleep. In support of our first aim, the intervention group exhibited significant in-session HRV increases compared to waitlist controls, confirming that the digital mindfulness application effectively induces short-term physiological relaxation. In parallel, several objective sleep metrics—such as sleep efficiency, total sleep, and light sleep—improved immediately after the 10-day intervention, with most of these benefits persisting at a 4-week follow-up (with the exception of deep sleep, which returned to baseline levels).

The significant improvements observed in sleep onset time are particularly noteworthy, as difficulties initiating sleep represent one of the most common complaints among individuals with sleep disturbances^14^. Our findings suggest that mindfulness practice may facilitate the transition from wakefulness to sleep by reducing pre-sleep cognitive arousal and rumination. This aligns with previous research demonstrating that mindfulness-based interventions can effectively address sleep onset latency by promoting a deactivation of the sympathetic nervous system^10,11^. The persistence of these improvements at the 4-week follow-up indicates that even a brief mindfulness intervention may produce lasting changes in sleep initiation patterns, potentially by establishing new pre-sleep cognitive habits that replace maladaptive rumination with present-moment awareness.

The observed increases in sleep efficiency and total sleep time following mindfulness training have significant implications for overall health. Sleep efficiency—the ratio of total sleep time to time spent in bed—is a critical metric of sleep quality, with higher efficiency associated with better restorative sleep^17^. The mindfulness group’s improved sleep efficiency suggests that participants not only fell asleep more readily but also experienced fewer nighttime awakenings, resulting in more consolidated sleep. This outcome is consistent with the findings of^9^, who demonstrated in their meta-analysis that mindfulness interventions can increase sleep efficiency by an average of 6.1% compared to control conditions. Our results further align with those of Black et al.^22^, whose randomized clinical trial found significant improvements in sleep efficiency following mindfulness training in older adults with sleep disturbances. Similarly, Gong et al.^23^ reported in their meta-analysis that mindfulness meditation produced moderate effects on sleep efficiency across diverse populations. Enhanced sleep efficiency and increased total sleep time have been linked to numerous health benefits, including improved immune function^24^, better cognitive performance^25^, and reduced risk for metabolic and cardiovascular disorders^26^.

The differential effects of mindfulness on specific sleep stages merit particular attention. Our findings revealed that light sleep significantly increased following mindfulness training, with benefits maintained at follow-up. Light sleep (NREM stages N1 and N2) plays essential roles in memory consolidation and cognitive processing^27^. The increase in light sleep proportion may reflect improved sleep continuity and more efficient sleep architecture. Conversely, although deep sleep (NREM stage N3) initially improved post-intervention, these benefits were not sustained at follow-up. Deep sleep is crucial for physical restoration and glymphatic clearance in the brain^28^. The transient nature of deep sleep improvements suggests that continued mindfulness practice may be necessary to maintain effects on this sleep stage, which is particularly vulnerable to age-related decline and stress^29^.

Notably, our study found no significant changes in REM sleep duration. This selective effect on non-REM sleep stages suggests that mindfulness may primarily influence slow-wave brain activity rather than REM-associated processes. These stage-specific findings contribute to our understanding of how mindfulness affects distinct neurophysiological mechanisms underlying sleep architecture. Future research should investigate whether extended mindfulness practice or different mindfulness techniques could produce more substantial and sustained effects on deep sleep and potentially influence REM sleep parameters.

A novelty of this study was the integration of digital health technology by combining mindfulness with ŌURA wearables. The use of the Ōura ring enabled continuous, objective monitoring of both physiological and sleep parameters in free-living conditions. Coupled with the in-app mindfulness intervention, this approach demonstrates the potential of digital therapeutics to not only deliver accessible stress-reduction techniques but also to capture rich, real-time data on autonomic regulation and sleep patterns. The observed link between acute HRV increases during mindfulness sessions and subsequent sleep improvements suggests that transient reductions in physiological arousal may facilitate smoother sleep transitions and better sleep maintenance. This effect might be driven by reduced cognitive arousal—such as decreased rumination and emotional reactivity^6,7^—and possibly by downregulating the hypothalamic–pituitary–adrenal axis, thereby lowering cortisol levels^30^.

Our analysis of practice timing provides important insights into the mechanisms by which mindfulness influences sleep. Although the majority of participants (58%) practiced in the evening (7–10 PM), sensitivity analyses revealed that sleep improvements were independent of practice timing, with morning and midday practitioners showing equivalent benefits. This finding suggests that mindfulness enhances sleep quality through mechanisms that operate throughout the day rather than requiring proximal timing to sleep onset. Specifically, participants who practiced primarily in the morning showed comparable improvements in sleep onset time as those practicing in the evening. This pattern indicates that the benefits likely accumulate through reduced cumulative stress exposure and improved emotion regulation across waking hours, which then facilitate the transition to sleep regardless of when practice occurred. This interpretation aligns with the broader stress-buffering effects of mindfulness documented by^30^ and supports the view articulated by^31^ that mindfulness creates a more favorable physiological foundation for sleep hrough its sustained effects on autonomic regulation and cognitive-emotional processing throughout the day. Even when practiced earlier in the day, these autonomic effects may create a physiological foundation for improved sleep that night by attenuating the cumulative impact of daily stressors on sleep-interfering hyperarousal^10^.

The significant increase in self-reported personal burnout (CBI1) immediately following the intervention presents a paradox: objective sleep metrics improved, yet participants reported feeling more burned out. Several interpretations merit consideration. First, mindfulness practice is known to increase interoceptive awareness and meta-cognitive monitoring^32,33^. Participants may have become more conscious of pre-existing fatigue and burnout symptoms that were previously operating below their threshold of awareness rather than experiencing an actual worsening of burnout. This heightened awareness might initially manifest as elevated self-reported symptoms before participants develop the regulatory skills to manage these perceptions effectively. Second, the 10-day intervention period may represent an initial adjustment phase—where increased self-awareness temporarily elevates symptom reporting before longer-term adaptive benefits consolidate. Supporting this interpretation, CBI1 scores showed a trend toward returning to baseline at the 4-week follow-up (though not statistically significant), suggesting the elevation was transient. However, we acknowledge this pattern could also indicate that brief mindfulness interventions insufficient to address burnout symptoms, or that paradoxically, increased awareness without adequate coping skill development may temporarily amplify distress. However, we must also consider alternative explanations. The finding could indicate that brief mindfulness interventions are insufficient to address burnout symptoms adequately, or that paradoxically, increased awareness without sufficient coping skill development may temporarily amplify distress. The dissociation between improved objective sleep metrics and increased subjective burnout also highlights the complex, non-linear relationship between physiological improvements and psychological well-being, suggesting these domains may operate on different timescales or through different mechanisms.

This unexpected finding requires cautious interpretation and warrants further investigation in studies with longer intervention periods, extended follow-ups, and qualitative assessments to clarify the mechanisms underlying this pattern.

Moreover, while the mindfulness intervention produced significant changes in objective measures, other self-reported evaluations such as the Perceived Stress Scale (PSS), Pittsburgh Sleep Quality Index (PSQI), and the work-related burnout subscale (CBI2) did not exhibit significant changes. This disparity suggests that short-term interventions may be more effective in eliciting immediate physiological responses than in altering longer-term subjective perceptions of stress and sleep quality. Future studies might consider lengthening the intervention period to determine whether more extended mindfulness practice yields significant changes in these subjective domains, thereby providing a more comprehensive understanding of both the acute and enduring effects of mindfulness.

The discrepancy between objective sleep improvements and minimal changes in subjective sleep quality (PSQI) deserves further consideration. This phenomenon—sometimes referred to as "sleep state misperception"—has been documented in previous mindfulness studies^9^, where objective sleep parameters improve before subjective awareness catches up. One explanation may be that the 10-day intervention period, while sufficient to induce physiological changes, was too brief to shift entrenched subjective perceptions of sleep quality. Alternatively, the heightened body awareness that typically develops through mindfulness practice may initially lead to greater sensitivity to sleep disturbances, temporarily overshadowing actual improvements in sleep^34^. This highlights the importance of combining objective and subjective measures in sleep research and suggests that longer interventions may be necessary to align subjective experiences with objective improvements.

The clinical implications of our findings extend beyond sleep improvements to potential applications in sleep disorder treatment. Currently, pharmacological interventions remain the predominant treatment for insomnia and other sleep disorders, despite concerns regarding side effects, dependency, and increased mortality risk^35–37^. Our results suggest that digital mindfulness interventions could serve as effective non-pharmacological alternatives or adjuncts, particularly for sleep onset difficulties and poor sleep efficiency. The observed improvements in these parameters are comparable to those reported for pharmacological interventions but without associated risks. Furthermore, the maintenance of effects at 4-week follow-up indicates that even brief mindfulness training may produce sustainable benefits, potentially through establishing enduring changes in pre-sleep cognitive patterns and autonomic regulation.

Our study is not without limitations. In addition to the noted discrepancies in self-reported outcomes, we did not control for potential confounding factors such as alcohol or substance use, which are known to affect both HRV and sleep quality^38,39^. Furthermore, the relatively short duration of the intervention and the sample’s composition—healthy adults with low self-reported stress—may have limited the scope of detectable changes and restricted the generalizability of our findings to more clinically relevant population. In addition, while sleep/wake classification accuracy is generally high for healthy individuals using consumer wearables, sleep-stage classification accuracy is more limited compared to polysomnography, which should be considered when interpreting our sleep findings. A significant methodological limitation was our use of a waitlist control design rather than an active control condition. An active control group would have been preferable to isolate mindfulness-specific effects from general relaxation or expectancy effects. The waitlist design limits our ability to determine whether the observed improvements in sleep metrics and physiological measures were specifically attributable to mindfulness practice. This represents a limitation of our intervention effects and should be addressed in future studies through the inclusion of appropriate active control conditions. We also note a limitation in the unbalanced sample sizes (n = 49 mindfulness vs. n = 32 waitlist control) resulting from differential attrition which may have influenced our ability to detect statistical significance across groups. While the waitlist control group showed improvements in some sleep metrics (e.g., total sleep time and sleep efficiency), these changes did not reach statistical significance. This pattern could reflect either genuine absence of intervention effects in the control group or insufficient statistical power due to the smaller sample size to detect changes of similar magnitude. Additionally, our study did not control for potential weekend/weekday effects on sleep patterns in the initial study design. A post-hoc analysis revealed a significant main effect of day-of-week, with participants sleeping approximately 23 min longer on weekends than weekdays. While our primary group × time interactions remained significant when controlling for day-of-week, future studies should incorporate this factor in the initial design, particularly given the high proportion of student participants whose sleep schedules may differ substantially between weekdays and weekends. We also acknowledge that the differential distribution of students across groups (higher proportion in mindfulness group) could have influenced results, though our sensitivity analysis restricted to student participants showed comparable effect sizes to the full sample. Additionally, we did not systematically collect qualitative feedback from participants about their adherence to specific mindfulness guidance within sessions or their subjective experiences during practice. Such feedback could have provided valuable insights into the mechanisms underlying the observed effects and helped explain discrepancies between objective and subjective outcomes. Future studies would benefit from incorporating mixed-methods approaches that combine quantitative metrics with qualitative assessments of participants’ engagement with and experiences of the intervention.

Future research should aim to address these limitations by incorporating longer intervention durations, broader and more diverse populations, and tighter control over potential confounders. Additionally, exploring the dose–response relationship of mindfulness practice could reveal whether more prolonged or frequent sessions lead to more pronounced improvements in both objective and subjective outcomes. Including qualitative feedback could also help clarify the nuanced relationship between self-perceived burnout and physiological changes. Future studies should also investigate whether targeting specific sleep parameters requires tailored mindfulness approaches. For example, while our general “destress” mindfulness exercise produced broad improvements in sleep, interventions specifically designed to address deep sleep might incorporate elements that directly target slow-wave activity, such as body scan techniques that have been shown to promote parasympathetic dominance^40^. Additionally, examining the potential synergistic effects of combining mindfulness with other behavioral sleep interventions, such as sleep restriction or stimulus control, could yield more comprehensive treatment protocols for clinical populations with more severe sleep disturbances.

In summary, our findings underscore the promise of integrating digital therapeutics with wearable health monitoring to deliver accessible mindfulness interventions. The observed improvements in in-session HRV and sleep metrics highlight the potential for these digital approaches to induce both immediate and lasting physiological benefits. However, discrepancies in self-reported measures and several study limitations call for more comprehensive future investigations to fully elucidate the relationship between mindfulness, physiological regulation, and perceived stress and sleep quality.

## Data Availability

The datasets generated during and/or analyzed during the current study are available from the corresponding author on reasonable request.

## References

[1] Walker, W. H., Walton, J. C., DeVries, A. C. & Nelson, R. J. Circadian rhythm disruption and mental health. *Transl. Psychiatry* **10**(1), 28. 10.1038/s41398-020-0694-0 (2020). 32066704 10.1038/s41398-020-0694-0 PMC7026420

[2] Freeman, D., Sheaves, B., Waite, F., Harvey, A. G. & Harrison, P. J. Sleep disturbance and psychiatric disorders. *Lancet Psychiatry* **7**(7), 628–637. 10.1016/S2215-0366(20)30136-X (2020). 32563308 10.1016/S2215-0366(20)30136-X

[3] Wirz-Justice, A., Bromundt, V. & Cajochen, C. Circadian disruption and psychiatric disorders: The importance of entrainment. *Sleep Med. Clin.* **4**(2), 273–284. 10.1016/j.jsmc.2009.01.008 (2009).

[4] Epstein, L. J. Sleep disorders. In *Sleep Disorders: Types and Approach to Evaluation* 2nd edn, Vol. 1 (ed. Cappucino, F. P.) 54–64 (Oxford University Press, 2018). 10.1093/oso/9780198778240.003.0007.

[5] Kerkhof, G. A. Epidemiology of sleep and sleep disorders in The Netherlands. *Sleep Med.* **30**, 229–239. 10.1016/j.sleep.2016.09.015 (2017). 28215254 10.1016/j.sleep.2016.09.015

[6] Jain, S. et al. A randomized controlled trial of mindfulness meditation versus relaxation training: Effects on distress, positive states of mind, rumination, and distraction. *Ann. Behav. Med.* **33**(1), 11–21. 10.1207/s15324796abm3301_2 (2007). 17291166 10.1207/s15324796abm3301_2

[7] Desbordes, G. et al. Effects of mindful-attention and compassion meditation training on amygdala response to emotional stimuli in an ordinary, non-meditative state. *Front. Hum. Neurosci.* **6**, 23050. 10.3389/fnhum.2012.00292 (2012). 10.3389/fnhum.2012.00292 PMC3485650 23125828

[8] Staiano, W. et al. Assessment of an App-based sleep program to improve sleep outcomes in a clinical insomnia population: Randomized controlled trial. *JMIR Mhealth Uhealth* **13**, e68665. 10.2196/686654 (2025). 40267472 10.2196/68665 PMC12059489

[9] Rusch, H. L. et al. The effect of mindfulness meditation on sleep quality: A systematic review and meta‐analysis of randomized controlled trials. *Ann. N. Y. Acad. Sci.* **1445**(1), 5–16. 10.1111/nyas.13996 (2019). 30575050 10.1111/nyas.13996 PMC6557693

[10] Ong, J. C. et al. A randomized controlled trial of mindfulness meditation for chronic insomnia. *Sleep* **37**(9), 1553–1563. 10.5665/sleep.4010 (2014). 25142566 10.5665/sleep.4010 PMC4153063

[11] Garland, S. N. et al. Mindfulness-based stress reduction compared with cognitive behavioral therapy for the treatment of insomnia comorbid with cancer: A randomized, partially blinded, noninferiority trial. *J. Clin. Oncol.* **32**(5), 449–457. 10.1200/JCO.2012.47.7265 (2014). 24395850 10.1200/JCO.2012.47.7265

[12] Kirk, U. & Axelsen, J. L. Heart rate variability is enhanced during mindfulness practice: A randomized controlled trial involving a 10-day online-based mindfulness intervention. *PLoS ONE* **15**(12), e0243488. 10.1371/journal.pone.0243488 (2020). 33332403 10.1371/journal.pone.0243488 PMC7746169

[13] Balsam, D., Bounds, D. T., Rahmani, A. M. & Nyamathi, A. Evaluating the impact of an app-delivered mindfulness meditation program to reduce stress and anxiety during pregnancy: Pilot longitudinal study. *JMIR Pediatr. Parent.* **6**, e53933. 10.2196/53933 (2023). 38145479 10.2196/53933 PMC10775027

[14] Ohayon, M. M. Epidemiological overview of sleep disorders in the general population. *Sleep Med. Res.* **2**(1), 1–9. 10.17241/smr.2011.2.1.1 (2011).

[15] Horne, J. A. & Ostberg, O. A self-assessment questionnaire to determine morningness-eveningness in human circadian rhythms.. *Int. J. Chronobiol.* **4**(2), 97–110 (1976). 1027738

[16] Cohen, S., Kamarck, T. & Mermelstein, R. A global measure of perceived stress. *J. Health Soc. Behav.* **24**(4), 385. 10.2307/2136404 (1983). 6668417

[17] Buysse, D. J., Reynolds, C. F., Monk, T. H., Berman, S. R. & Kupfer, D. J. The Pittsburgh sleep quality index: A new instrument for psychiatric practice and research. *Psychiatry Res.* **28**(2), 193–213. 10.1016/0165-1781(89)90047-4 (1989). 2748771 10.1016/0165-1781(89)90047-4

[18] Kristensen, T. S., Borritz, M., Villadsen, E. & Christensen, K. B. The Copenhagen burnout inventory: A new tool for the assessment of burnout. *Work Stress* **19**(3), 192–207. 10.1080/02678370500297720 (2005).

[19] Brown, K. W. & Ryan, R. M. The benefits of being present: Mindfulness and its role in psychological well-being.. *J. Pers. Soc. Psychol.* **84**(4), 822–848. 10.1037/0022-3514.84.4.822 (2003). 12703651 10.1037/0022-3514.84.4.822

[20] Li, J. et al. Sleep duration and health outcomes: An umbrella review. *Sleep Breath* **26**(3), 1479–1501. 10.1007/s11325-021-02458-1 (2022). 34435311 10.1007/s11325-021-02458-1

[21] Smith, M. T. et al. Use of actigraphy for the evaluation of sleep disorders and circadian rhythm sleep-wake disorders: An American Academy of Sleep Medicine clinical practice guideline. *J. Clin. Sleep Med.* **14**, 1231–1237. 10.5664/jcsm.7230 (2018). 29991437 10.5664/jcsm.7230 PMC6040807

[22] Black, D. S., O’Reilly, G. A., Olmstead, R., Breen, E. C. & Irwin, M. R. Mindfulness meditation and improvement in sleep quality and daytime impairment among older adults with sleep disturbances. *JAMA Intern. Med.* **175**(4), 494. 10.1001/jamainternmed.2014.8081 (2015). 25686304 10.1001/jamainternmed.2014.8081 PMC4407465

[23] Gong, H. et al. Mindfulness meditation for insomnia: A meta-analysis of randomized controlled trials. *J. Psychosom. Res.* **89**, 1–6. 10.1016/j.jpsychores.2016.07.016 (2016). 27663102 10.1016/j.jpsychores.2016.07.016

[24] Irwin, M. et al. Partial night sleep deprivation reduces natural killer and celhdar immune responses in humans. *FASEB J.* **10**(5), 643–653. 10.1096/fasebj.10.5.8621064 (1996). 8621064 10.1096/fasebj.10.5.8621064

[25] Walker, M. P. & Stickgold, R. Sleep-dependent learning and memory consolidation. *Neuron* **44**(1), 121–133. 10.1016/j.neuron.2004.08.031 (2004). 15450165 10.1016/j.neuron.2004.08.031

[26] Wolk, R., Gami, A., Garciatouchard, A. & Somers, V. Sleep and cardiovascular disease. *Curr. Probl. Cardiol.* **30**(12), 625–662. 10.1016/j.cpcardiol.2005.07.002 (2005). 16301095 10.1016/j.cpcardiol.2005.07.002

[27] Walker, M. P., Brakefield, T., Morgan, A., Hobson, J. A. & Stickgold, R. Practice with sleep makes perfect. *Neuron* **35**(1), 205–211. 10.1016/S0896-6273(02)00746-8 (2002). 12123620 10.1016/s0896-6273(02)00746-8

[28] Jessen, N. A., Munk, A. S. F., Lundgaard, I. & Nedergaard, M. The glymphatic system: A beginner’s guide. *Neurochem. Res.* **40**(12), 2583–2599. 10.1007/s11064-015-1581-6 (2015). 25947369 10.1007/s11064-015-1581-6 PMC4636982

[29] Krause, A. J. et al. The sleep-deprived human brain. *Nat. Rev. Neurosci.* **18**(7), 404–418. 10.1038/nrn.2017.55 (2017). 28515433 10.1038/nrn.2017.55 PMC6143346

[30] Garland, E. L., Hanley, A. W., Baker, A. K. & Howard, M. O. Biobehavioral mechanisms of mindfulness as a treatment for chronic stress: An RDoC perspective. *Chronic Stress* **1**, 1–15. 10.1177/2470547017711912 (2017). 10.1177/2470547017711912 PMC5565157 28840198

[31] Tang, Y.-Y. et al. Short-term meditation training improves attention and self-regulation. *Proc. Natl. Acad. Sci. U.S.A.* **104**(43), 17152–17156. 10.1073/pnas.0707678104 (2007). 17940025 10.1073/pnas.0707678104 PMC2040428

[32] Kirk, U., Gu, X., Harvey, A. H., Fonagy, P. & Montague, P. R. Mindfulness training modulates value signals in ventromedial prefrontal cortex through input from insular cortex. *Neuroimage* **100**, 254–262. 10.1016/j.neuroimage.2014.06.035 (2014). 24956066 10.1016/j.neuroimage.2014.06.035 PMC4140407

[33] Garland, E. L., Farb, N. A., Goldin, P. R. & Fredrickson, B. L. The mindfulness-to-meaning theory: Extensions, applications, and challenges at the attention–appraisal–emotion interface. *Psychol. Inq.* **26**(4), 377–387. 10.1080/1047840X.2015.1092493 (2015).

[34] de Vibe, M. et al. Six-year positive effects of a mindfulness-based intervention on mindfulness, coping and well-being in medical and psychology students; results from a randomized controlled trial. *PLoS ONE* **13**(4), e0196053. 10.1371/journal.pone.0196053 (2018). 29689081 10.1371/journal.pone.0196053 PMC5916495

[35] Kripke, D. F., Langer, R. D. & Kline, L. E. Hypnotics’ association with mortality or cancer: A matched cohort study. *BMJ Open* **2**(1), e000850. 10.1136/bmjopen-2012-000850 (2012). 22371848 10.1136/bmjopen-2012-000850 PMC3293137

[36] O’Donnell, M. L. et al. Impact of the diagnostic changes to post-traumatic stress disorder for DSM-5 and the proposed changes to ICD-11. *Br. J. Psychiatry* **205**(3), 230–235. 10.1192/bjp.bp.113.135285 (2014). 24809400 10.1192/bjp.bp.113.135285

[37] Brower, K. J. et al. Prescription sleeping pills, insomnia, and suicidality in the National Comorbidity Survey Replication. *J. Clin. Psychiatry* **72**(04), 515–521. 10.4088/JCP.09m05484gry (2011). 20868634 10.4088/JCP.09m05484gry

[38] Ralevski, E., Petrakis, I. & Altemus, M. Heart rate variability in alcohol use: A review. *Pharmacol. Biochem. Behav.* **176**, 83–92. 10.1016/j.pbb.2018.12.003 (2019). 30529588 10.1016/j.pbb.2018.12.003

[39] Sjoberg, N. & Saint, D. A. A single 4 mg dose of nicotine decreases heart rate variability in healthy nonsmokers: Implications for smoking cessation programs. *Nicotine Tob. Res.* **13**(5), 369–372. 10.1093/ntr/ntr004 (2011). 21350044 10.1093/ntr/ntr004

[40] Liu, Y.-L., Lee, C.-H. & Wu, L.-M. A mindfulness-based intervention improves perceived stress and mindfulness in university nursing students: A quasi-experimental study. *Sci. Rep.* **14**(1), 13220. 10.1038/s41598-024-64183-5 (2024). 38851820 10.1038/s41598-024-64183-5 PMC11162462

